# Tomato leaf disease detection based on attention mechanism and multi-scale feature fusion

**DOI:** 10.3389/fpls.2024.1382802

**Published:** 2024-04-09

**Authors:** Yong Wang, Panxing Zhang, Shuang Tian

**Affiliations:** School of Computer Science and Technology, Zhejiang University of Technology, Hangzhou, China

**Keywords:** disease detection, YOLOv6, attention mechanism, feature pyramid network, multi-scale feature fusion

## Abstract

When detecting tomato leaf diseases in natural environments, factors such as changes in lighting, occlusion, and the small size of leaf lesions pose challenges to detection accuracy. Therefore, this study proposes a tomato leaf disease detection method based on attention mechanisms and multi-scale feature fusion. Firstly, the Convolutional Block Attention Module (CBAM) is introduced into the backbone feature extraction network to enhance the ability to extract lesion features and suppress the effects of environmental interference. Secondly, shallow feature maps are introduced into the re-parameterized generalized feature pyramid network (RepGFPN), constructing a new multi-scale re-parameterized generalized feature fusion module (BiRepGFPN) to enhance feature fusion expression and improve the localization ability for small lesion features. Finally, the BiRepGFPN replaces the Path Aggregation Feature Pyramid Network (PAFPN) in the YOLOv6 model to achieve effective fusion of deep semantic and shallow spatial information. Experimental results indicate that, when evaluated on the publicly available PlantDoc dataset, the model’s mean average precision (mAP) showed improvements of 7.7%, 11.8%, 3.4%, 5.7%, 4.3%, and 2.6% compared to YOLOX, YOLOv5, YOLOv6, YOLOv6-s, YOLOv7, and YOLOv8, respectively. When evaluated on the tomato leaf disease dataset, the model demonstrated a precision of 92.9%, a recall rate of 95.2%, an F1 score of 94.0%, and a mean average precision (mAP) of 93.8%, showing improvements of 2.3%, 4.0%, 3.1%, and 2.7% respectively compared to the baseline model. These results indicate that the proposed detection method possesses significant detection performance and generalization capabilities.

## 1 Introduction

Tomatoes are widely cultivated globally and are a significant source of income for many agricultural countries. Due to various environmental factors such as climate change, tomatoes are highly susceptible to infections by fungi, bacteria, and viruses, which severely affect their yield and quality. Early detection and identification of these diseases are crucial for reducing the infection and spread among tomato plants, with initial symptoms often appearing on the leaves. Therefore, accurate disease identification becomes crucial (Yao et al., 2023). Traditional disease detection methods rely on the experiential judgment of agricultural experts, which are not only inefficient but also limited in accuracy, unable to meet the needs of modern high-efficiency agriculture. With the development of computer and Internet of Things (IoT) technologies, integrating object detection technology into tomato production has become an important trend in the modernization of tomato cultivation.

The powerful autonomous learning capability of deep learning has enhanced neural network performance, becoming a major trend and new direction in agricultural disease detection (Sunil et al., 2023). Compared to traditional machine learning, deep learning-based object detection algorithms offer advantages such as faster detection speeds, higher accuracy, and better generalization capabilities (Liu and Wang, 2021). Currently, the mainstream object detection algorithms include classic models such as Faster R-CNN (Ren et al., 2015), SSD (Liu et al., 2016), and YOLO series (Ge et al., 2021; Li et al., 2022a; Wang et al., 2023). Consequently, researchers have conducted a series of detection tasks under experimental conditions, demonstrating the potential of object detection algorithms in agricultural disease detection. Albattah et al. (2021) proposed a framework for automatic detection and classification of plant diseases based on DenseNET-77, which cannot be deployed on mobile devices. Jing et al. (2023)improved upon YOLOv5 by incorporating the Convolutional Block Attention Module (CBAM) and replacing the original FPN with BiFPN, achieving detection of 9 types of tomato diseases and healthy leaves with a 96.4% accuracy rate while also obtaining good tomato disease image annotation results. However, data images in controlled experimental environments often have simple backgrounds and sufficient lighting. When dealing with diseases in complex environments, the detection performance is inadequate and difficult to meet the requirements of actual production environments.

In the natural environment, factors such as lighting, soil conditions, and climate pose many challenges for leaf disease detection, such as difficulties in disease localization due to lighting changes, feature loss from obstruction, similarity of different disease symptoms, and small lesion sizes. Faced with these challenges, researchers have proposed methods for leaf disease detection in natural environments. Roy et al. (2022) proposed a high-performance, fine-grained object detection framework based on YOLOv4, addressing issues of irregular shapes, multi-scale targets, and similar textures in plant disease detection. Li et al. (2022c) proposed a lightweight detection method that combines improved YOLOv5 and ShuffleNet to detect peach tree leaf diseases in natural environments, albeit with a slight decrease in accuracy. Liu and Wang (2023) introduced a mixed attention mechanism into the feature prediction structure of YOLOv5 to improve the detection of tomato brown spot disease in complex scenes. Zhang et al. (2022) improved the real-time detection of cotton pests and diseases in complex natural environments by introducing efficient channel attention (ECA), hard-Swish activation function, and Focal Loss function based on the YOLOX model. Liu et al. (2023) proposed PKAMMF based on the YOLOv7 model to address the challenges of complex natural backgrounds, indistinct disease features, and partial obstruction. This method integrates a prior knowledge attention mechanism, adding new feature fusion layers and prediction layers to enhance the detection capability of small objects. On their custom tomato disease dataset, the mean Average Precision (mAP) reached 91.96%, but the addition of fusion layers reduced inference speed. Qi et al. (2022) proposed a tomato virus disease detection method based on SE-YOLOv5, incorporating the SE attention mechanism to extract key disease features, thus enhancing the detection accuracy of tomato diseases. Li et al. (2022b) proposed a multi-scale detection method for cucumber diseases in natural scenes. This method combines CA and Transformer to reduce interference from irrelevant background information, and employs a multi-scale training strategy to enhance the detection of small lesions. Researchers (Guo et al., 2022; Zhao et al., 2022; Cai and Jiang, 2023) also from the perspectives of multi-scale feature fusion concepts and attention mechanisms, achieve the detection of diseases in the leaves of grapes, strawberries, and other plants in complex environments.

Although previous detection methods have achieved certain results in leaf disease, accurately identifying tomato leaf diseases in real environments remains challenging. The shadows created by changes in lighting affect the characterization of disease lesions on tomato leaves, making them resemble the spots caused by tomato leaf mold. Early blight and septoria leaf spot in tomatoes initially show small, roughly circular spots with relatively few texture features, leading to a high incidence of misdiagnosis. Given these common yet challenging issues in complex natural environments, the accuracy of existing methods for detecting tomato leaf diseases needs to be improved. The aforementioned studies have shown that adding P2 fusion layers and additional small-scale detection heads can improve the accuracy of detecting small lesions, but also comes with a higher computational cost. Furthermore, by introducing attention mechanisms at different positions within the model structure, the model places more emphasis on the weighted learning of lesion features, further enhancing its performance in detecting leaf diseases. However, it is necessary to select the appropriate attention mechanism based on the characteristics of the leaf diseases.

The YOLO series is widely used for real-time detection of agricultural diseases due to its excellent balance between speed and accuracy, among which the YOLOv6 model, considering real-world conditions, has advantages in balancing inference speed and detection precision. Therefore, this paper selects this model as the baseline model. However, the model has some limitations in processing images of diseases with small leaf lesions and natural environmental factors such as lighting and occlusion. Therefore, addressing the aforementioned issues and based on the aforementioned research findings, this study proposes an improved tomato leaf disease detection method based on the YOLOv6 model, with the main contributions as follows:

(1) The Convolutional Block Attention Module (CBAM) is integrated into the backbone feature extraction network of the model, refining the feature maps from both channel and spatial dimensions, thereby emphasizing the weighted learning of features in tomato leaf lesions.
(2) The Reparameterized Generalized Feature Pyramid Network (RepGFPN) was improved by introducing small-scale features and developing a new feature fusion module(BiRepGFPN), which captured the characteristics of small lesions on tomato leaves.
(3) By replacing the original feature fusion network with BiRepGFPN, shallow spatial information and deep semantic information are efficiently aggregated, achieving precise localization of tomato leaf lesions at various scales.

## 2 Materials and Methods

### 2.1 Data Acquisition

Current studies on disease detection predominantly concentrate on identifying lesions. This study considers that in actual agricultural production, lesions often affect the growth and health condition of the entire leaf, and lesion shapes are diverse. Therefore, this paper selects the entire leaf as the research subject, aiming to conduct disease detection from a global perspective. The dataset utilized in this study originates from the publicly accessible tomato leaf disease dataset on the roboflow1 platform (Bryan, 2023; SREC, 2023; projectdesign, 2023), comprising the PlantVillage dataset, the PlantDoc (Singh et al., 2020) dataset, internet-captured data, and photographs taken by the researchers themselves. PlantVillage includes 14 different crops, such as potatoes, tomatoes, apples, soybeans, sweet peppers, etc., with a total of 54,309 images. The tomato segment features images of 9 diseases and 1 healthy state. However, the images in PlantVillage are captured under laboratory settings, missing the context of real-world environments. The PlantDoc dataset contains 30 categories, including 13 plant species and 17 disease types, with the tomato leaf disease categories consistent with PlantVillage. The images in PlantDoc were collected in various natural environments and feature multiple annotation boxes within a single image.

This paper selects several common but difficult to accurately identify tomato leaf diseases from the original dataset to form a new tomato leaf disease dataset. The characteristics of the dataset are as follows.

(1) Complex lighting and occlusion conditions: The dataset accounts for occlusion between leaves, as well as shadows and light spots caused by changes in lighting. Additionally, the data includes occlusions caused by external objects such as soil.
(2) Diversity of disease features: The dataset contains variations of lesions throughout the disease lifecycle, covering different sizes, shapes, textures, and colors.
(3) Dense overlapping among leaves: Considering the challenges in real agricultural environments, the dataset includes phenomena of dense overlapping among leaves, which increases the complexity of detection.

**Figure 1:**
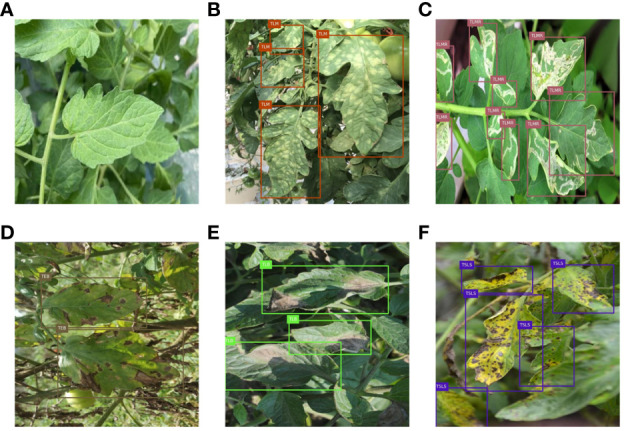
Sample images of tomato leaf diseases: **(A)** healthy leaves; **(B)** tomato leaf mold; **(C)** tomato leaf miner; **(D)** tomato early blight; **(E)** tomato late light; **(F)** tomato septoria leaf spot.

The dataset includes tomato septoria leaf spot (TSLS), tomato early blight (TEB), tomato leaf mold (TLM), tomato late light (TLB), tomato leaf miner (TLMR), and healthy leaves (TL), totaling 1425 images. Considering that healthy leaves are not detected in actual disease detection, annotations for healthy leaves were removed. The dataset was randomly divided into a training set and a test set at a ratio of 8:2. To ensure precise assessment of model performance, training and test sets were kept distinct without any overlap, and data in each category were uniformly distributed following an identical ratio. Sample images of tomato leaf diseases are shown in **Figure 1**. It can be observed that tomato early blight and tomato septoria leaf spot exhibit relatively small lesions on the leaves, and there is similarity between these two diseases, making them difficult to distinguish. The symptoms of tomato leaf miner can easily be confused with the texture of healthy leaves. The lesions of tomato leaf mold and tomato late blight are susceptible to the effects of lighting.

### 2.2 Data Preprocessing

To increase the diversity of training samples, this paper applied data augmentation to the training set, including random noise addition, brightness adjustment, and flipping, ultimately expanding the training set to 4659 images. The detailed information of the training set is shown in **Table 1**. **Table 1** lists the number of each disease category in the original training set and the augmented training set.

**Table 1:** Tomato leaf disease training set distribution.

Diseases	Before augmentation	After augmentation
tomato leaf miner	263	1125
tomato early blight	234	945
tomato late light	182	713
tomato leaf mold	159	673
tomato septoria leaf spot	108	424
healthy leaves	189	779

**Figure 2:**
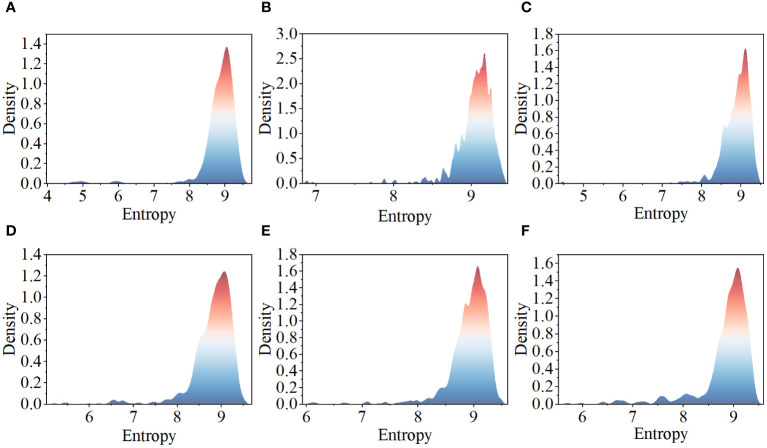
Tomato leaf disease image complexity distribution: **(A)** healthy leaves; **(B)** tomato leaf mold; **(C)** tomato leaf miner; **(D)** tomato late light; **(E)** tomato early blight; **(F)** tomato septoria leaf spot.

This paper assesses the complexity of images by calculating their entropy values (Wu et al., 2022), with high entropy values typically indicating that the images contain more textures and objects. **Figure 2** displays the complexity distribution of images from each category in the tomato disease dataset using a density plot. The x-axis represents the entropy values of images, with higher entropy values indicating greater complexity. The y-axis indicates the probability density values, with higher values suggesting a greater number of images near that entropy value. Observing the distribution in the figure, it is found that the complexity of images from each category is mostly concentrated near high entropy values, indicating that the images in this dataset have a certain level of complexity and richness of information. This analysis aids in gaining a deeper understanding of the characteristics of this dataset.

### 2.3 YOLOv6

The YOLOv6 network architecture is divided into four parts: the input layer, backbone network, feature fusion network, and detection head. The input layer performs data augmentation, such as Mosaic, and size-adaptive scaling on the input images before feeding them into the backbone network. The backbone network, also known as the feature extraction network, consists of a series of re-parameterizable (Ding et al., 2021) convolutions (RepConv), residual blocks (CSPRepBlock), and spatial pyramids (SimSPPF), mainly for extracting feature information of various scales. A notable feature of this network is the design of an efficient re-parameterizable backbone (EfficientRep), which fully utilizes the computing capabilities of hardware, significantly reducing inference latency and enhancing feature representation ability.

The feature fusion network employs a Path Aggregation Feature Pyramid Network structure (PAFPN) for the fusion of multi-scale features from the output of the backbone network, facilitating interaction among features at different levels. By adopting CSPRepBlock, it maintains good multi-scale feature fusion capability while ensuring efficient inference. The feature fusion network outputs feature maps of sizes 80×80, 40×40, and 20×20, to detect small, medium, and large objects, respectively. The detection head employs a Hybrid Channels strategy to construct a more efficient decoupled head, which reduces latency while maintaining accuracy. This module predicts outcomes for feature maps of three different scales, thereby obtaining the final object location and category information.

### 2.4 Improved YOLOv6

While the YOLOv6 model has achieved significant results in general object detection, it has some limitations in detecting tomato leaf diseases in natural environments. Due to its inability to effectively suppress interference information when dealing with environmental factors such as lighting and occlusion, the YOLOv6 model has certain limitations in the effectiveness of distinguishing features. Additionally, the fusion method in the YOLOv6 model feature fusion network does not fully consider deep semantic information and shallow spatial information. Moreover, the features covered by the feature maps of three scales are relatively limited, making it weaker in capturing characteristics of small leaf spots.

**Figure 3:**
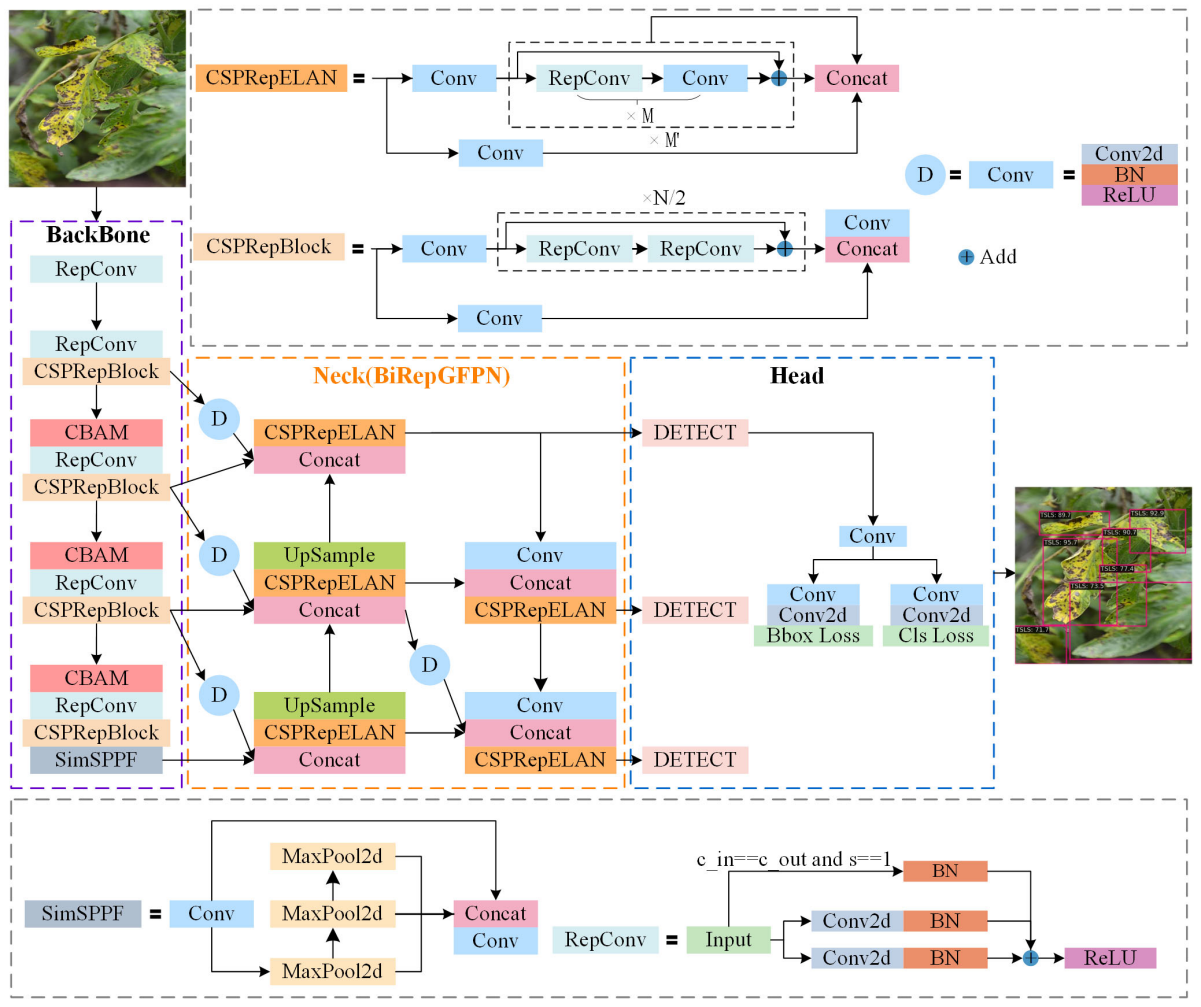
Improved YOLOv6 network structure diagram.

Considering the aforementioned limitations, this study aims to improve the original YOLOv6 model in two aspects. Firstly, by integrating CBAM (Woo et al., 2018) following three CSPRepBlocks in the backbone network of YOLOv6, the approach suppresses redundant information from complex backgrounds and enhances the capability to extract disease spot features. Secondly, a multi-scale reparametrized generalization feature fusion module(BiRepGFPN), is proposed to enhance the expressive power of feature fusion by aggregating shallow features, thereby improving the accuracy of disease localization at different scales. The network architecture of the improved YOLOv6 model is illustrated in **Figure 3**.

#### 2.4.1 Convolutional Block Attention Module

**Figure 4:**
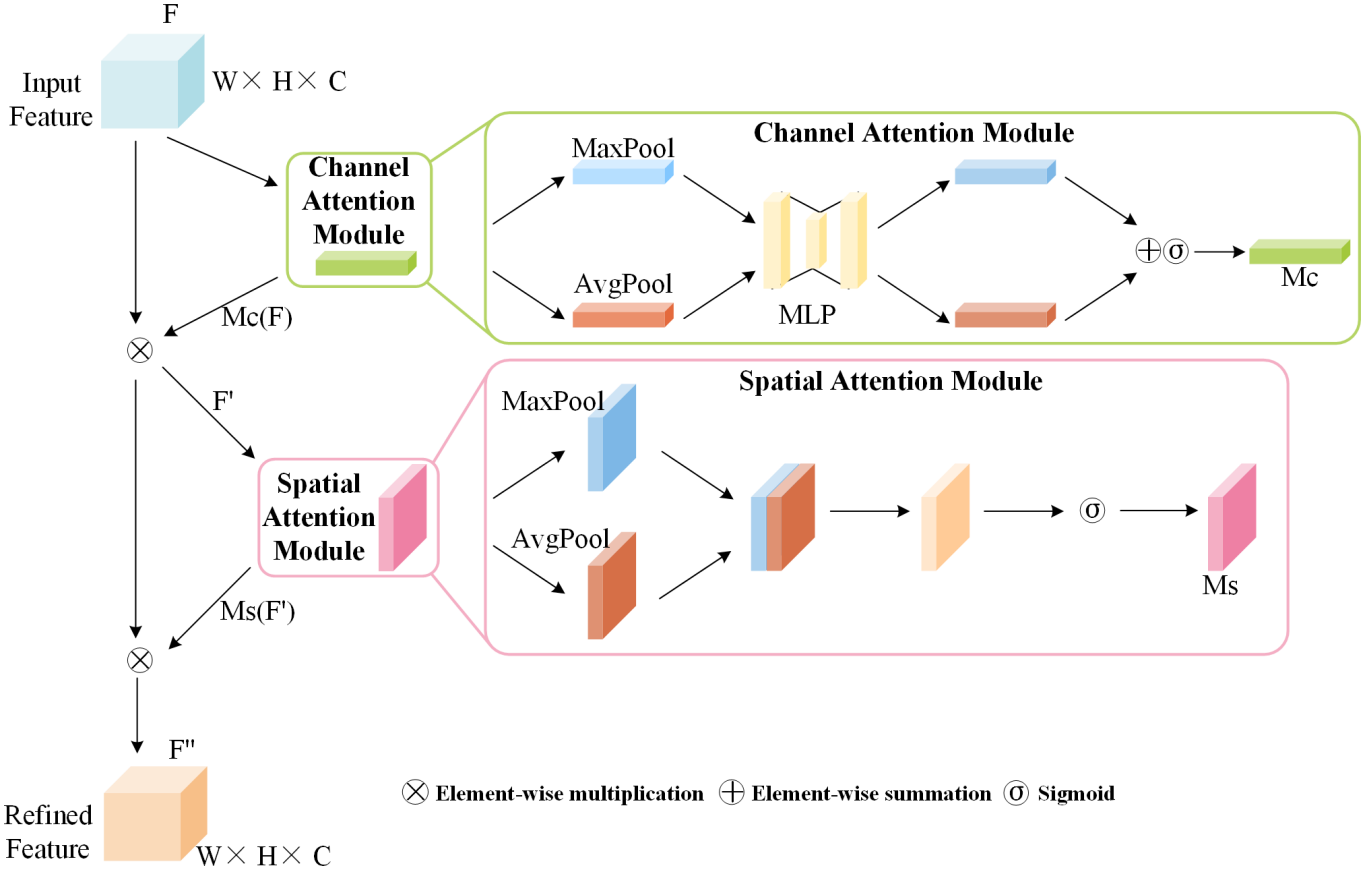
CBAM network structure diagram.

The baseline model YOLOv6, when processing image feature maps, is insensitive to differences in image features, thus exhibiting poor suppression of redundant information brought about by complex environments. This study considers that the spatial dimensions of feature maps contain spatial location information of diseases, with each channel containing different features. Consequently, the CBAM attention mechanism was introduced, emphasizing the importance of channels and spatial dimensions by allocating more weight to key channel information and spatial location information. As a data processing method, CBAM can enhance key features through autonomous learning while suppressing irrelevant background information. As shown in **Figure 4**, CBAM is a hybrid attention mechanism that combines spatial and channel aspects.

The channel attention module of CBAM concentrates on channels, applying global average pooling and global max pooling separately to the input features $F\in R^{W\times H\times C}$ to produce two novel feature maps. These two feature maps are fed into a three-layer neural network multilayer perceptron (MLP), and after element-wise summation, the channel attention map $M_{c}\left(F\right)$ is obtained through a Sigmoid activation function. $M_{c}\left(F\right)$ is multiplied by the feature map F to obtain the stage output feature map, with the calculation formula of $M_{c}\left(F\right)$ as shown in Equation 1:


(1) Mc (F)=σ(MLP(AvgPool(F))+MLP(MaxPool(F)))

The spatial attention module focuses on meaningful positional information within the feature map. It takes the feature map $F^{\prime }$ output by the channel attention module as input. This input is then subjected to max pooling and average pooling, followed by concatenation along the channel dimension. The spatial attention map $M_{s}\left(F^{\prime }\right)$ is obtained through a 7×7 convolution operation followed by a Sigmoid activation function. $M_{s}\left(F^{\prime }\right)$ is multiplied by the feature $F^{\prime }$ to obtain the final output feature map $F^{\prime \prime }$ , with the calculation formula for $M_{s}\left(F^{\prime }\right)$ as shown in Equation 2:


(2) $$M_{s}\left(F^{\prime }\right)=\sigma \left(conv^{7\times 7}\left(\left[AvgPool\left(F^{\prime }\right);MaxPool\left(F^{\prime }\right)\right]\right)\right)$$

In the formula, σ represents the Sigmoid activation function, and $conv^{7\times 7}$ denotes convolution operations with a kernel size of 7×7.

#### 2.4.2 Multi-Scale Reparameterization Generalized Feature Fusion Module

**Figure 5:**
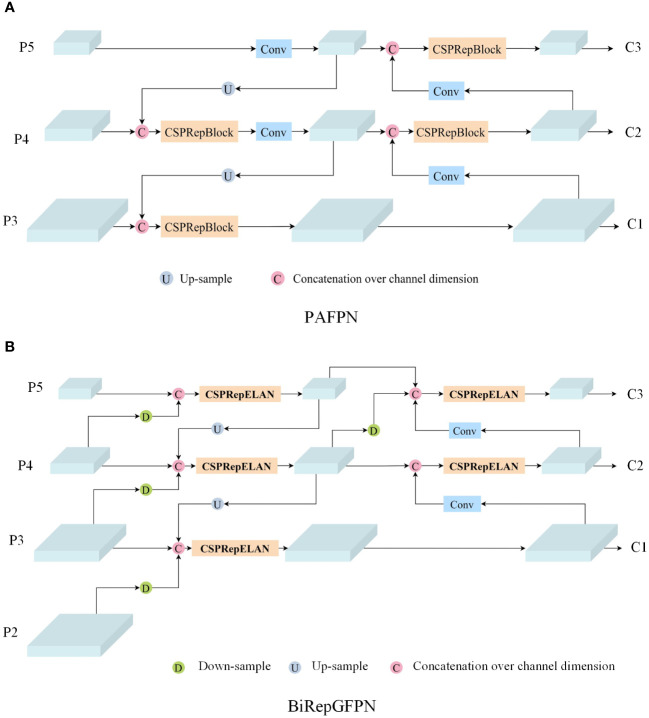
Feature fusion network structure diagram: **(A)** PAFPN; **(B)** BiRepGFPN.

The symptoms of tomato leaf diseases are diverse and vary in size, posing certain challenges for disease detection. Feature Pyramid Networks (FPN) can effectively detect objects of different sizes. FPN primarily relies on hierarchical pyramid features, building a top-down feature fusion pathway to progressively transfer deep features to shallow levels, achieving the fusion of features at different levels. However, during this process, the deep features are not fused with the shallow features. To further enhance feature transmission, Path Aggregation Feature Pyramid Network (PAFPN) introduces a bottom-up path based on FPN, allowing deep features to extract finer information from shallow features, thereby improving the accuracy of location features. The structural diagram of PAFPN is shown in **Figure 5A**, where P3, P4, P5 represent the output feature maps obtained by the backbone network downsampling the input image by 8, 16, and 32 times, respectively.C1, C2, C3 represent the three output feature maps of the fusion network, respectively. Nonetheless, PAFPN can only support top-down and bottom-up feature fusion, with a relatively simple method of fusion.

The YOLOv6 model originally employed PAFPN as its feature fusion network. This network merges feature maps of adjacent levels through the transfer of information between them, without fully considering the effective integration across different levels of feature maps. However, as feature maps at different levels each contain unique information, a more refined fusion strategy can achieve effective complementarity and enhancement between them. Therefore, to enhance the model’s ability to understand image details and structures, and to more comprehensively capture the scale changes and complexity of leaf disease spots, this study employs the efficient layer aggregation network RepGFPN (Xu et al., 2022), which features skip connections and cross-scale links, as the feature fusion network. RepGFPN is an improvement on the Generalized Feature Pyramid Network (GFPN). GFPN (Tan et al., 2021) employs Queen-Fusion for enhanced feature fusion, which strengthens feature representation capabilities. However, due to the sharing of the same number of channels across different scales, there is feature redundancy, and the extensive use of upsampling and downsampling operations results in low inference efficiency. RepGFPN optimizes both the method of feature fusion and the network structure. Firstly, it employs varying numbers of channels for features at different scales, flexibly controlling the expressive capabilities of deep and shallow features. Secondly, a cross-stage partial network (CSPNet) with reparameterization concepts and efficient layer aggregation network (ELAN) connections was introduced, aimed at merging features of adjacent layers and different scales within the same level. The network structure as shown in the CSPRepELAN module in **Figure 3**. The shallow layers of the feature extraction network typically contain richer spatial information, while the deep layers have richer semantic information. Therefore, to more precisely locate the features of small lesions on tomato leaves, this study uses the feature map P2, downsampled by a factor of 4 from the feature extraction network, as a new output. P2 is then downsampled and fused with the P3 feature map for enhanced feature integration. This improvement aims to enhance the fusion of deep semantic information and shallow spatial information, thereby more effectively detecting tomato leaf diseases at various scales. The improved Feature Pyramid Network is referred to as BiRepGFPN, with the network schematic shown in **Figure 5B**.

## 3 Results

### 3.1 Experimental Environment

All experiments in this paper were conducted in the same hardware and software environment. The main parameters of the training platform used for this experiment are: NVIDIA RTX A6000, 48GB of VRAM, CUDA version 11.1, Ubuntu 18.04.5 LTS operating system, and PyTorch 1.9.0 as the deep learning framework. During the training process, the initial learning rate was set to 0.01, batch size to 16, number of epochs to 300, using the SGD optimizer. To save computational resources, training was conducted using mixed precision and early stopping strategies.

### 3.2 Evaluation Metrics

This paper uses precision (P), recall (R), average precision (AP50), F1 score, and mean average precision (mAP50) as the evaluation metrics for the model. Precision represents the proportion of correctly predicted samples among the samples predicted to be of the positive class by the model. Recall represents the proportion of correctly predicted samples among the samples that are actually of the positive class. The F1 score is the harmonic mean of precision and recall, reflecting the balance between precision and recall and avoiding extreme values in either metric. The calculation formulas are as shown in Equations 3–5:


(3) $$P=\frac{TP}{TP+FP}$$

(4) $$R=\frac{TP}{TP+FN}$$

(5) $$F1\;score=\frac{2\times P\times R}{P+R}$$

In the formula, TP represents the number of samples correctly predicted as positive. FP represents the number of samples incorrectly predicted as positive. FN represents the number of samples that are actually positive but were incorrectly predicted as negative. The PR curve, formed with recall on the horizontal axis and precision on the vertical axis, represents precision and recall at different thresholds. The area under the PR curve is defined as the AP, reflecting the performance of target detection accuracy, and the mAP is the mean of AP across multiple categories. AP50 represents the average precision for a certain category at a specific IoU threshold of 0.5, and mAP50 represents the average precision across multiple categories at the same IoU threshold of 0.5. The calculation formulas are as shown in Equations 6, 7:


(6) $$AP50=\int_{0}^{1}P\left(r\right)dr,IoU\geq 0.5$$

(7) $$mAP50=\frac{\sum_{i=0}^{N}AP50_{i}}{N},IoU\geq 0.5$$

### 3.3 Results and Analysis

#### 3.3.1 Comparative Analysis of Attention Mechanisms

To verify the impact of different attention mechanisms on the YOLOv6 model, this paper incorporates the CBAM, Efficient Channel Attention ECA (Wang et al., 2020), Coordinate Attention CA (Hou et al., 2021), and Efficient Multi-Scale Attention EMA (Ouyang et al., 2023) into the YOLOv6 model for experimentation.

**Figure 6:**
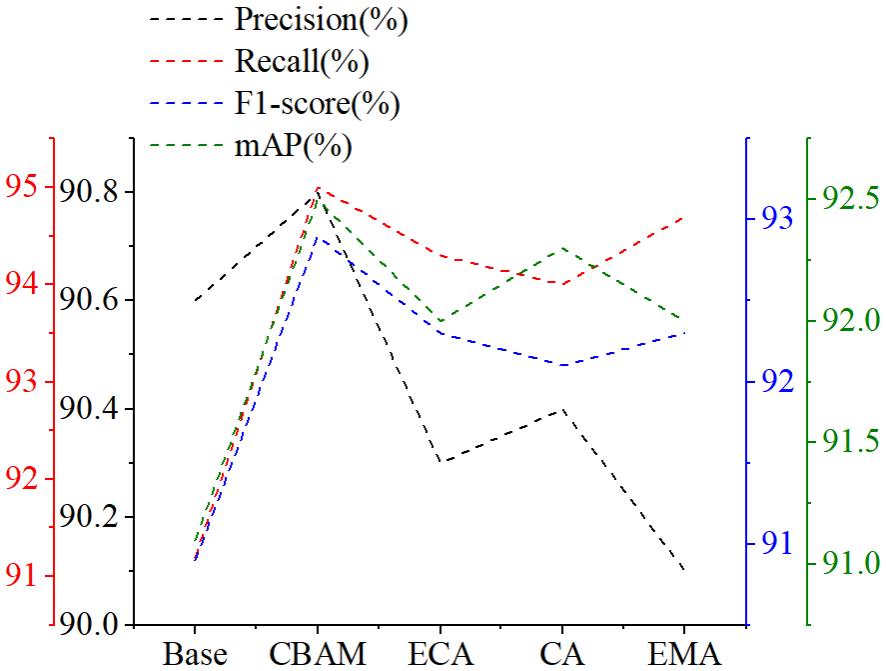
Comparison of different attention mechanisms: Base represents the YOLOv6.

By analyzing the data results in **Figure 6**, it was found that the introduction of attention mechanisms had a positive impact on enhancing the overall performance of the model. Among the four popular attention mechanisms compared, CBAM showed superior performance in the metrics of precision, recall, F1 score, and mAP, outperforming the other attention mechanisms.

ECA introduces deformable convolutions to capture the relationships between channels, while CA enhances feature maps by learning the importance of each channel. However, both primarily focus on channel information. EMA integrates features of CA and convolution, aiming to learn multi-scale features and proposes a cross-space information aggregation method for richer feature integration. Nevertheless, the cross-space learning of EMA may introduce noise. CBAM, by integrating channel attention and spatial attention, fully leverages the information of channels and space, effectively suppressing interference from complex backgrounds, and thus is more conducive to the learning of tomato leaf lesion features.

**Figure 7:**
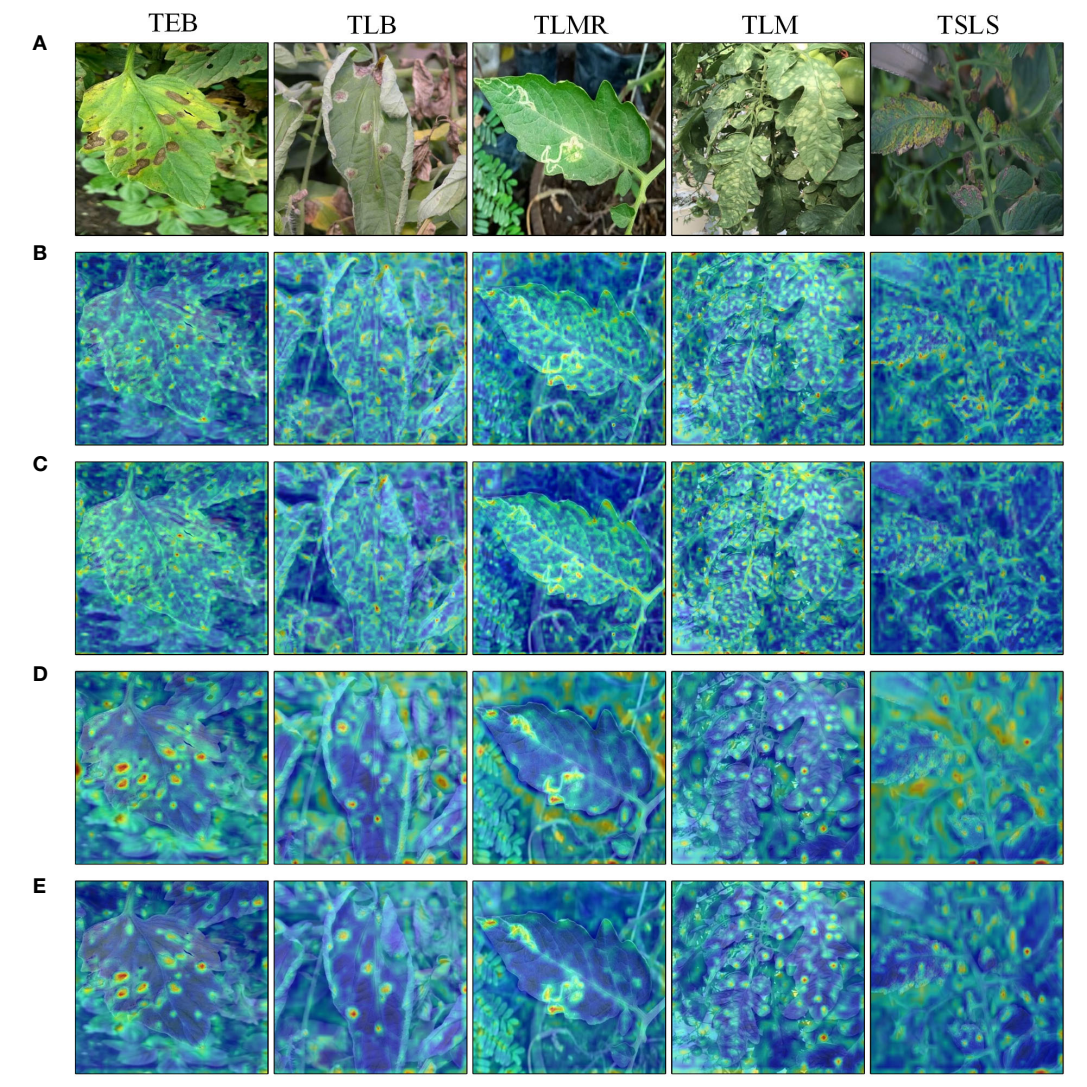
Feature map visualization: **(A)** Original image; **(B)** YOLOv6 shallow feature map; **(C)** Shallow feature map after introducing CBAM; **(D)** YOLOv6 intermediate feature map; **(E)** Intermediate feature map after introducing CBAM.

**Figure 7** visualizes certain hierarchical feature maps output by the backbone network through heatmaps, intuitively demonstrating the effectiveness of CBAM in suppressing redundant information and filtering key features. Deep red in the figure indicates areas of increased focus by the network, while deep blue indicates lesser attention. Analysis of the heatmaps reveals that the YOLOv6 model is prone to interference from the background during the detection process, making it difficult for the network to effectively extract key feature information of leaf lesions. Comparing heatmaps before and after incorporating the CBAM module reveals that the introduction of the attention mechanism allows the model to effectively suppress background influence, focusing more on the weight learning of leaf lesion features. In the figure, the feature extraction effects for the tomato early blight, tomato late light, tomato leaf miner, and tomato leaf mold are evident. However, for septoria leaf spot, due to its less distinct, dense, and relatively small presence on the leaves, the introduction of the attention mechanism did not enable the model to effectively distinguish between the key features of the leaf lesions and the redundant background information.

Experimental results show that by integrating CBAM with YOLOv6, the model focuses more on the features of leaf lesions, reducing the learning of redundant information in the background. However, the model’s ability to recognize densely distributed and relatively small lesion features on leaves is still limited. To address this issue, further enhancement of model detection capability for densely distributed and small lesion features is necessary.

#### 3.3.2 Analysis of the Effectiveness of BiRepGFPN

**Figure 8:**
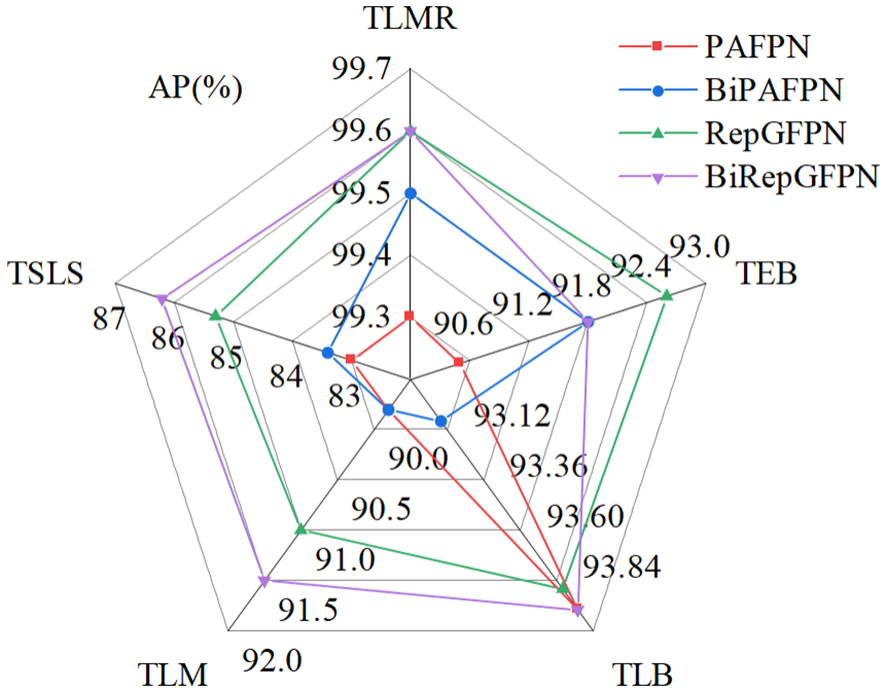
Comparison of different feature fusion methods.

**Figure 9:**
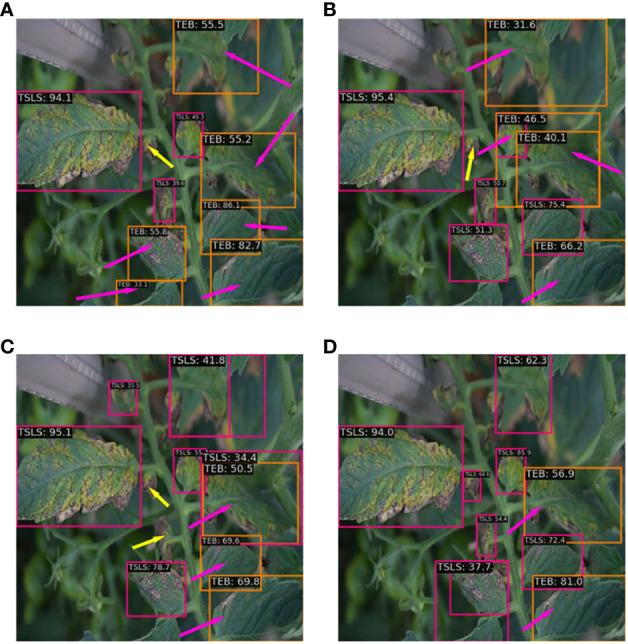
The detection effects of different fusion networks: **(A)** PAFPN; **(B)** BiPAFPN; **(C)** RepGFPN; **(D)** BiRepGFPN.

To assess detection performance of the feature pyramid network on various types of tomato leaf disease, this study employed four different feature fusion methods to train and test on the tomato leaf disease dataset, including PAFPN, BiPAFPN, RepGFPN, and BiRepGFPN. Among these, BiPAFPN represents an attempt made in this paper to introduce a P2 layer on the basis of PAFPN, and to downsample the P2 layer for fusion with the P3 layer. The related experimental results are shown in **Figures 8**, **9**.

The experimental data from **Figure 8** indicate that modifications to the feature pyramid network enhanced the detection of tomato leaf diseases. The study found that the simple bidirectional feature fusion method used in PAFPN could not effectively integrate shallow and deep feature information. In contrast, the use of multipath feature fusion methods, such as BiPAFPN, RepGFPN, and BiRepGFPN, significantly improved the interaction and integration of information between feature maps of different scales. Additionally, the introduction of the finer-grained shallow feature map P2 increased the accuracy of disease detection. In the detection of the tomato leaf miner, the average precision of BiRepGFPN reached 99.6%.

In the detection of the tomato leaf mold and tomato early blight, BiRepGFPN showed an improvement of 1.7% and 3.2% in average precision over PAFPN, respectively. However, in the detection of the tomato late light, BiRepGFPN had a slight decrease in average precision by 0.8% compared to RepGFPN, possibly due to the redundancy brought about by the introduction of the P2 layer. Nonetheless, BiRepGFPN still demonstrated a 1.3% improvement over PAFPN. Considering the detection performance across five diseases, BiRepGFPN holds certain advantages in handling different scale features of tomato leaf diseases. **Figure 9** displays the detection results of four feature pyramid fusion networks on minor spots of tomato leaves. Red arrows indicate false detections, and yellow arrows mark missed detections. It can be observed from the figure that multipath feature fusion methods such as BiPAFPN, RepGFPN, and BiRepGFPN demonstrate superior detection performance for small lesions compared to the simple bidirectional feature fusion method PAFPN. Among these, the combination of BiRepGFPN with YOLOv6 achieved the best detection performance, effectively integrating feature map information of different scales, enhancing the feature extraction and localization ability for small lesions, and achieving the lowest false positive rate.

#### 3.3.3 Ablation Experiment

To evaluate the impact of the improved methods proposed in this study on the performance of tomato leaf disease detection, a series of ablation experiments were conducted. These experiments aim to test the effectiveness of each improvement individually, thereby clarifying the contribution of each improvement to the overall performance enhancement. The experimental results are shown in **Table 2**.

**Table 2:** Results of ablation experiments.

YOLOv6	CABM	BiRepGFPN	Precision (%)	Recall (%)	F1 score (%)	mAP (%)
√			90.6	91.2	90.9	91.1
√	√		90.8	95.0	92.9	92.5
√		√	90.1	97.0	93.4	92.6
√	√	√	92.9	95.2	94.0	93.8

The above experimental results reveal:

(1) Compared to the baseline model, after introducing the CBAM attention mechanism into the backbone network, the model showed varying degrees of improvement in precision, recall, F1 score, and mAP. This result indicates that the attention mechanism effectively suppresses the interference of redundant information in complex environments by allocating weights in both spatial and channel dimensions, thereby enhancing the model’s detection performance.
(2) Compared to the baseline model, the improvement method of BiRepGFPN had a positive effect on model performance, especially in terms of significant improvements in recall, F1 score, and mAP, although there was a slight decrease in precision. It is analyzed that, on one hand, the interaction between feature maps of different layers might introduce some noise, thus affecting the accuracy of detection. On the other hand, the significant increase in recall underscores the importance of integrating shallow spatial information with deep semantic information, which reduces the miss rate of leaf disease but also leads to a certain degree of false positives.
(3) In comparing the two improvement strategies, it was found that CBAM and BiRepGFPN each have their advantages in terms of precision and recall, respectively. Among them, BiRepGFPN performed better in balancing precision and recall.

Overall, this study leverages the strengths of two distinct improvement strategies. Compared to the baseline model, precision, recall, F1 score, and mAP increased by 2.3%, 4.0%, 3.1%, and 2.7%, respectively. These results validate the effectiveness of the proposed methods and demonstrate the potential in detecting tomato leaf diseases in complex environments.

#### 3.3.4 Comparison of Mainstream Model Performance

To validate improved model detection performance on tomato leaf diseases in complex environments and generalizability to diseases of other crops, this paper conducts training and testing on tomato leaf disease dataset, PlantDoc dataset and FieldPlant (Moupojou et al., 2023) dataset. Given the high speed and accuracy of the YOLO series in real-time detection, YOLOX, YOLOv5, YOLOv6, YOLOv7, YOLOv8 (Jocher et al., 2023) and improved YOLOv6 were selected for comparative experiments. The tomato leaf disease dataset focuses on tomato leaf diseases in complex environments, while the PlantDoc dataset includes not only tomato leaf diseases but also diseases of other crops, thus verifying the generalizability of the improved model.

The detection results on the tomato leaf disease dataset are shown in **Table 3**. As can be observed from **Table 3**, compared to the original YOLOv6 model, the improved model has seen increases of 3.1% in F1 score, 4.0% in recall, 2.3% in precision, and 2.7% in the mAP. Although its precision is slightly inferior to other models, it has the highest recall rate. This is because a higher recall rate can reduce precision to some extent. Taking into account various evaluation criteria, compared to the other six models, the improved model in this paper achieves the best balance between precision and recall rate, reaching the highest mAP.

**Table 3:** Performance indicators of different models in the tomato leaf disease dataset.

Model	Precision (%)	Recall (%)	F1 score (%)	mAP (%)
YOLOX	92.6	87.2	89.8	90.5
YOLOv5	93.5	78.0	85.0	85.6
YOLOv6	90.6	91.2	90.9	91.1
YOLOV6-s	88.9	91.3	90.1	89.1
YOLOv7	94.9	84.2	89.2	88.6
YOLOv8	91.5	90.3	90.9	89.8
Ours	92.9	95.2	94.0	93.8

**Figure 10:**
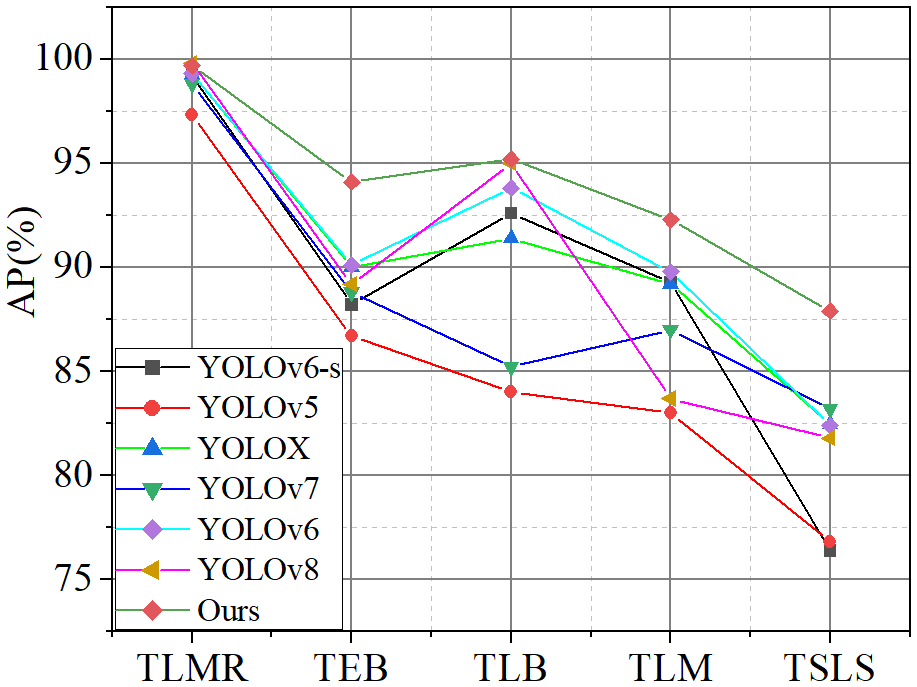
Disease detection performance of different models.

**Figure 10** shows the average precision for each disease by seven models in detecting diseases on the tomato leaf disease dataset. It can be seen from the figure that the improvements in this paper achieved an accuracy rate of over 90% in detecting the tomato leaf miner, tomato early blight, tomato late light, and tomato leaf mold. In the detection of the tomato septoria leaf spot, the model demonstrated the highest accuracy compared to other models.

In the detection of tomato leaf miner, due to its distinct disease characteristics from other diseases, especially in terms of texture clarity, the model is able to recognize it with a high degree of accuracy. For tomato late blight in tomatoes, the relatively large size of the lesions enables the model to also demonstrate high accuracy in detecting this disease. In handling tomato leaf mold, the distinct color of the lesions from other diseases provides a good basis for differentiation by the model. However, in detecting tomato early blight and tomato late blight, the main challenges arise from the small size of the lesions and the difficulty in capturing texture information, coupled with a certain degree of similarity in the shapes of these two diseases, which increases the difficulty of feature extraction. Notably, this paper significantly improves the accuracy of detecting small lesions by implementing fusion between feature maps of different levels.

In summary, the improved method proposed in this paper demonstrates significant effectiveness in the detection of tomato leaf diseases. This is not only reflected in the high accuracy of detecting diseases with clear characteristics (such as tomato leaf miner, tomato late blight, and tomato leaf mold) but also in the significant improvement in detecting performance for disease categories that are more difficult in feature extraction (such as tomato early blight and tomato late blight).

**Figure 11:**
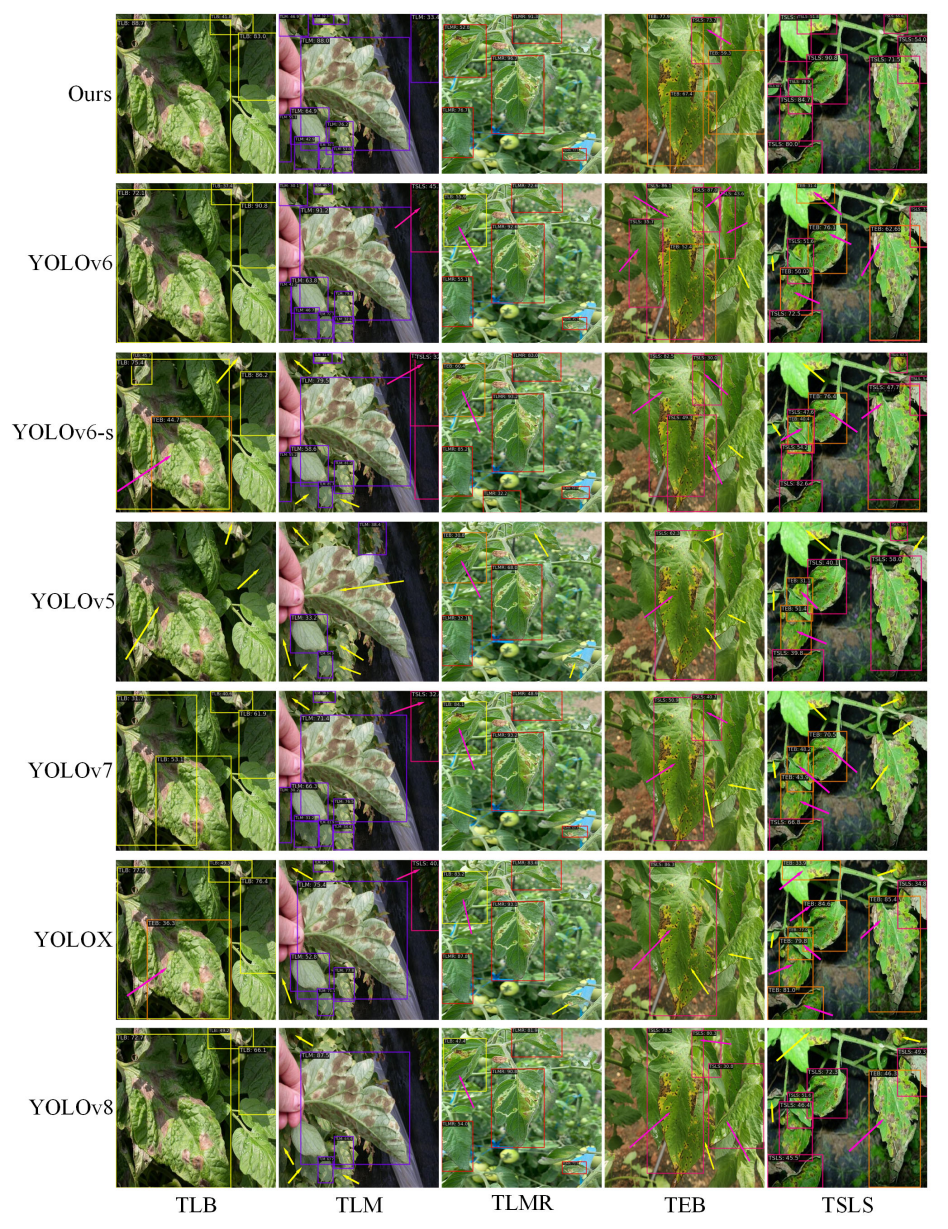
Comparison of prediction results from different models.

**Figure 11** illustrates the visualization of detection results by six models on the tomato leaf disease dataset. Red arrows indicate false detections, and yellow arrows denote missed detections. Observations from the first column reveal that under conditions of lighting and shadow interference, YOLOX and YOLOv6-s misidentify late blight as early blight, while YOLOv5 experiences severe missed detections. The reason for this phenomenon is that, when the lesions of tomato early blight and tomato late blight are slightly larger, the differences in shape and color are minimal. Coupled with the effect of lighting, texture information is further disturbed, leading to this misidentification. In complex scenes with multiple targets obstructing each other, as in columns 2 and 3, the improved model in this paper is the only one that can accurately identify the tomato leaf miner and tomato leaf mold, while other models suffer from missed and false detections. Specifically, the leaf wilting symptoms caused by the tomato leaf miner are similar to those of early blight and late blight, leading to the misidentification of the tomato leaf miner. For cases with densely distributed small lesions and occlusions, as shown in columns 4 and 5, all seven models exhibit varying degrees of false detection of tomato early blight and tomato septoria leaf spot. This is because both tomato early blight and tomato septoria leaf spot appear as small circles in the early stages of growth, making the expression of disease characteristics less obvious. Observing from the figures, YOLOv5, YOLOX, YOLOv7, and YOLOv8 exhibit poor performance in handling occlusions present in the images. When detecting the diverse lesion characteristics of tomato septoria leaf spot, YOLOv8 and the improved model presented in this paper demonstrate a detection advantage. However, in detecting the small lesions of tomato early blight, only the model improved in this paper achieved optimal precise detection. Therefore, compared to YOLOv5, YOLOX, YOLOv7, YOLOv6, YOLOv6-s, and YOLOv8, the enhanced YOLOv6 model exhibits superior performance in detecting and recognizing diseases of different scales in complex environments, demonstrating a significant overall performance advantage.

Most images in PlantDoc have low resolution and noticeable noise, making disease detection more challenging. The results from **Table 4** show that, in terms of F1 score, the proposed improved model has increased by 7.4%, 12.7%, 9.4%, 5.2%, 3.4%, and 2.4% compared to YOLOX, YOLOv5, YOLOv6, YOLOv6-s, YOLOv7, and YOLOv8, respectively. In terms of mAP, compared to YOLOX, YOLOv5, YOLOv6, YOLOv6-s, YOLOv7, and YOLOv8, the improved model has increased by 7.7%, 11.8%, 3.4%, 5.7%, 4.3%, and 2.6%, respectively. Overall, the model performs excellently on the PlantDoc dataset, achieving significant improvements in both F1 score and mAP compared to other models. This further validates model effectiveness on public datasets and demonstrates generalization ability in detecting diseases in other crops.

Images in the FieldPlant dataset directly come from plantations, featuring various lighting conditions, totaling 5,156 images. The dataset focuses on various diseases on leaves of corn, cassava, and tomatoes. Considering the high resolution of images in the dataset might lead to significant computational resource consumption, this study manually adjusted the resolution of images and the size of annotation boxes to reduce resource demands while trying to maintain image quality as much as possible. As can be seen from the results in **Table 4**, the method proposed in this paper shows a significant advantage over YOLOX, YOLOv5, YOLOv6-s, YOLOv7, and YOLOv8. However, compared to the YOLOv6 model, the F1 score is slightly higher, and the mAP is slightly lower. This performance discrepancy might stem from the disease annotation boxes in the FieldPlant dataset containing more background information, such as soil, weeds, and sky, due to the angle of photography, which leads to more redundant information in the disease annotation boxes for corn leaves. Additionally, the BiRepGFPN module proposed in this paper enhances feature fusion through connections between different levels of feature maps, but it may also introduce more noise, thereby affecting the detection performance. Therefore, future research could further improve the model, especially in terms of optimizing feature fusion and reducing noise interference.

**Table 4:** Performance indicators of different models in the PlantDoc dataset and FieldPlant dataset.

Dataset	Model	Precision (%)	Recall (%)	F1-score (%)	mAP (%)
PlantDoc	YOLOX	66.8	69.4	68.1	69.6
	YOLOv5	71.0	56.3	62.8	65.5
	YOLOv6	67.6	78.4	66.1	73.9
	YOLOv6-s	65.0	76.4	70.3	71.6
	YOLOv7	73.5	70.7	72.1	73.0
	YOLOv8	75.4	70.9	73.1	74.7
	Ours	69.0	83.4	75.5	77.3
FieldPlant	YOLOX	65.0	66.2	65.6	61.7
	YOLOv5	58.8	47.5	52.5	53.7
	YOLOv6	64.4	75.4	69.5	67.1
	YOLOv6-s	63.3	70.1	66.5	65.7
	YOLOv7	67.8	57.5	62.2	64.8
	YOLOv8	69.9	61.5	65.4	66.4
	Ours	70.1	69.6	69.8	66.5

## 4 Conclusion

To enhance the accuracy of tomato leaf disease detection in complex environments, this paper has improved the YOLOv6 model. By incorporating CBAM, the emphasis of the model on crucial disease features has been amplified, effectively diminishing environmental interference. Simultaneously, improvements were made on the basis of RepGFPN by proposing BiRepGFPN to replace the feature fusion network, enhancing the ability of the model to capture small lesion features and express less obvious characteristics. The research results indicate that the improved YOLOv6 model can precisely detect tomato leaf diseases in natural environments, demonstrating more effective performance compared to other commonly used models. On the public dataset PlantDoc, the model still achieved better detection results, further verifying its advantage in generalization performance.

However, due to the imbalanced distribution of disease features in the dataset, the model in this paper still has room for further improvement when dealing with small disease spots with similar symptoms. Furthermore, its performance on the public dataset FieldPlant requires further enhancement. Future research work includes:

(1) Improve and optimize the model structure: Optimize the design of the aggregated residual blocks to reduce information loss and noise amplification during the fusion process, thereby enhancing the model’s detection capabilities.
(2) Designing Rotated Annotation Boxes: Considering the actual shape of the leaves and the angle of photography, rotate the annotation boxes for leaf diseases to include more key information. The design of the rotated boxes should be reflected both in the dataset preprocessing and model design stages.
(3) Dataset diversification: Expand the dataset to include image data from different growth stages, seasons, and regions to enhance the model’s generalization ability and robustness.
(4) Multimodal data processing: Combine non-visual data, such as temperature and humidity, for multimodal recognition, to enhance the accuracy and robustness of disease detection in natural environments.

## Data availability statement

The original contributions presented in the study are included in the article/supplementary material. Further inquiries can be directed to the corresponding author/s.

## Author contributions

WY: Funding acquisition, Methodology, Project administration, Resources, Supervision, Writing – review & editing. ZX: Conceptualization, Data curation, Investigation, Methodology, Software, Validation, Writing – original draft. TS: Conceptualization, Formal analysis, Validation, Writing – review & editing.

## References

[B1] Albattah W. Nawaz M. Javed A. Masood M. Albahli S. (2021). A novel deep learning method for detection and classification of plant diseases. Complex Intelligent Syst. 8, 507–524. doi: 10.1007/s40747-021-00536-1

[B2] Bryan. (2023). Tomato leaf disease dataset [Open source dataset]. Roboflow Universe. Available at: https://universe.roboflow.com/bryan-b56jm/tomato-leaf-disease-ssoha.

[B3] Cai H. Jiang J. (2023). “An improved plant disease detection method based on YOLOv5,” in 2023 15th International Conference on Intelligent Human-Machine Systems and Cybernetics (IHMSC). (Hangzhou, China: IEEE), 237–240. doi: 10.1109/IHMSC58761.2023.00062

[B4] Ding X. Zhang X. Ma N. Han J. Ding G. Sun J. (2021). “Repvgg: Making vgg-style convnets great again,” in Proceedings of the IEEE/CVF conference on computer vision and pattern recognition. (Nashville, TN, USA: IEEE), 13733–13742. doi: 10.1109/CVPR46437.2021.01352

[B5] Ge Z. Liu S. Wang F. Li Z. Sun J. (2021). Yolox: Exceeding yolo series in 2021. arXiv [Preprint]. doi: 10.48550/arXiv.2107.08430

[B6] Guo W. Feng Q. Li X. Yang S. Yang J. (2022). ). Grape leaf disease detection based on attention mechanisms. Int. J. Agric. Biol. Eng. 15, 205–212. doi: 10.25165/j.ijabe.20221505.7548

[B7] Hou Q. Zhou D. Feng J. (2021). “Coordinate attention for efficient mobile network design,” in Proceedings of the IEEE/CVF conference on computer vision and pattern recognition. (Nashville, TN, USA: IEEE), 13708–13717. doi: 10.1109/CVPR46437.2021.01350

[B8] Jing J. Li S. Qiao C. Li K. Zhu X. Zhang L. (2023). A tomato disease identification method based on leaf image automatic labeling algorithm and improved YOLOv5 model. J. Sci. Food Agriculture. 103, 7070–7082. doi: 10.1002/jsfa.12793 37326973

[B9] Jocher G. Chaurasia A. Qiu J. (2023) “YOLO by Ultralytics”. Available online at: https://github.com/ultralytics/ultralytics (Accessed February 30, 2023).

[B10] Li C. Li L. Jiang H. Weng K. Geng Y. Li L.. (2022a). YOLOv6: A single-stage object detection framework for industrial applications. arXiv [Preprint]. doi: 10.48550/arXiv.2209.02976

[B11] Li S. Li K. Qiao Y. Zhang L. (2022b). A multi-scale cucumber disease detection method in natural scenes based on YOLOv5. Comput. Electron. Agric. 202, 107363. doi: 10.1016/j.compag.2022.107363

[B12] Li Y. Li A. Li X. Liang D. (2022c). “Detection and identification of peach leaf diseases based on YOLO v5 improved model,” in Proceedings of the 5th International Conference on Control and Computer Vision. (New York, NY, USA: ACM), 79–84. doi: 10.1145/3561613.3561626

[B13] Liu J. Wang X. (2021). Plant diseases and pests detection based on deep learning: a review. Plant Methods 17, 1–18. doi: 10.1186/s13007-021-00722-9 33627131 PMC7903739

[B14] Liu J. Wang X. (2023). Tomato disease object detection method combining prior knowledge attention mechanism and multiscale features. Front. Plant Sci. 14. doi: 10.3389/fpls.2023.1255119 PMC10590886 37877077

[B15] Liu J. Wang X. Zhu Q. Miao W. (2023). Tomato brown rot disease detection using improved YOLOv5 with attention mechanism. Front. Plant Sci. 14. doi: 10.3389/fpls.2023.1289464 PMC10694285 38053763

[B16] Liu W. Anguelov D. Erhan D. Szegedy C. Reed S. Fu C. Y.. (2016). “Ssd: Single shot multibox detector,” in European conference on computer vision. (Cham: Springer), 21–37. doi: 10.1007/978-3-319-46448-0_2

[B17] Moupojou E. Tagne A. Retraint F. Tadonkemwa A. Wilfried D. Tapamo H.. (2023). FieldPlant: A dataset of field plant images for plant disease detection and classification with deep learning. IEEE Access 11, 35398–35410. doi: 10.1109/ACCESS.2023.3263042

[B18] Ouyang D. He S. Zhan J. Guo H. Huang Z. Luo M.. (2023). “Efficient multi-scale attention module with cross-spatial learning,” in ICASSP 2023 - 2023 IEEE International Conference on Acoustics, Speech and Signal Processing (ICASSP). (Rhodes Island, Greece: IEEE), 1–5. doi: 10.1109/ICASSP49357.2023.10096516

[B19] projectdesign. (2023). Tomato biotic stress classification dataset [Open source dataset]. Roboflow Universe. Available at: https://universe.roboflow.com/projectdesign-rw5fo/tomato-biotic-stress-classification.

[B20] Qi J. Liu X. Liu K. Xu F. Guo H. Tian X.. (2022). An improved YOLOv5 model based on visual attention mechanism: Application to recognition of tomato virus disease. Comput. Electron. Agric. 194, 106780. doi: 10.1016/j.compag.2022.106780

[B21] Ren S. He K. Girshick R. B. Sun J. (2015). Faster R-CNN: towards real-time object detection with region proposal networks. IEEE Trans. Pattern Anal. Mach. Intell. 39, 1137–1149. doi: 10.1109/TPAMI.2016.2577031 27295650

[B22] Roy A. M. Bose R. Bhaduri J. (2022). A fast accurate fine-grain object detection model based on YOLOv4 deep neural network. Neural Computing Appl. 34, 3895–3921. doi: 10.1007/s00521-021-06651-x

[B23] Singh D. Jain N. Jain P. Kayal P. Kumawat S. Batra N. (2020). “PlantDoc: A dataset for visual plant disease detection,” in Proceedings of the 7th ACM IKDD CoDS and 25th COMAD. (New York, NY, USA: ACM), 249–253. doi: 10.1145/3371158.3371196

[B24] SREC. (2023). Early- dataset [Open source dataset]. Roboflow Universe. Available at: https://universe.roboflow.com/srec/early.

[B25] Sunil C. K. Jaidhar C. D. Patil N. (2023). Systematic study on deep learning-based plant disease detection or classification. Artif. Intell. Rev. 56, 14955–15052. doi: 10.1007/s10462-023-10517-0

[B26] Tan Z. Wang J. Sun X. Lin M. Li H. (2021) Giraffedet: A heavy-neck paradigm for object detection. In International conference on learning representations. Available online at: https://openreview.net/forum?id=cBu4ElJfneV.

[B27] Wang C. Y. Bochkovskiy A. Liao H. Y. M. (2023). “YOLOv7: Trainable bag-of-freebies sets new state-of-the-art for real-time object detectors,” in Proceedings of the IEEE/CVF Conference on Computer Vision and Pattern Recognition. (Vancouver, BC, Canada: IEEE), 7464–7475. doi: 10.1109/CVPR52729.2023.00721

[B28] Wang Q. Wu B. Zhu P. Li P. Zuo W. Hu Q. (2020). “ECA-Net: Efficient channel attention for deep convolutional neural networks,” in Proceedings of the IEEE/CVF conference on computer vision and pattern recognition. (Seattle, WA: IEEE), 11531–11539. doi: 10.1109/CVPR42600.2020.01155

[B29] Woo S. Park J. Lee J. Y. Kweon I. S. (2018). “Cbam: Convolutional block attention module,” in Proceedings of the European conference on computer vision (ECCV). (Cham: Springer International Publishing), Vol. 11211, 3–19. doi: 10.1007/978-3-030-01234-2_1

[B30] Wu J. Chen J. Huang D. (2022). “Entropy-based active learning for object detection with progressive diversity constraint,” in Proceedings of the IEEE/CVF Conference on Computer Vision and Pattern Recognition. (New Orleans, LA, USA: IEEE), 9397–9406. doi: 10.1109/CVPR52688.2022.00918

[B31] Xu X. Jiang Y. Chen W. Huang Y. Zhang Y. Sun X. (2022). Damo-yolo: a report on real-time object detection design. arXiv [Preprint]. doi: 10.48550/arXiv.2211.15444

[B32] Yao J. Tran S. N. Sawyer S. Garg S. (2023). Machine learning for leaf disease classification: data, techniques and applications. Artif. Intell. Rev. 56, 3571–3616. doi: 10.1007/s10462-023-10610-4

[B33] Zhang Y. Ma B. Hu Y. Li C. Li Y. (2022). Accurate cotton diseases and pests detection in complex background based on an improved YOLOX model. Comput. Electron. Agric. 203, 107484. doi: 10.1016/j.compag.2022.107484

[B34] Zhao S. Liu J. Wu S. (2022). Multiple disease detection method for greenhouse-cultivated strawberry based on multiscale feature fusion Faster R_CNN. Comput. Electron. Agric. 199, 107176. doi: 10.1016/j.compag.2022.107176

